# The design of novel inhibitors for treating cancer by targeting CDC25B through disruption of CDC25B-CDK2/Cyclin A interaction using computational approaches

**DOI:** 10.18632/oncotarget.16600

**Published:** 2017-03-27

**Authors:** Hong-Lian Li, Ying Ma, Ying Ma, Yu Li, Xiu-Bo Chen, Wei-Li Dong, Run-Ling Wang

**Affiliations:** ^1^ Tianjin Key Laboratory on Technologies Enabling Development of Clinical Therapeutics and Diagnostics (Theranostics), School of Pharmacy, Tianjin Medical University, Tianjin, China; ^2^ Eye Hospital, Tianjin Medical University, School of Optometry and Ophthalmology, Tianjin Medical University, Tianjin, China

**Keywords:** CDC25B, ZDOCK, RDOCK, replace fragment, molecular dynamic simulation

## Abstract

Cell division cycle 25B is a key cell cycle regulator and widely considered as potent clinical drug target for cancers. This research focused on identifying potential compounds in theory which are able to disrupt transient interactions between CDC25B and its CDK2/Cyclin A substrate.

By using the method of ZDOCK and RDOCK, the most optimized 3D structure of CDK2/Cyclin A in complex with CDC25B was constructed and validated using two methods: 1) the superimposition of proteins; 2) analysis of the hydrogen bond distances of Arg 488(N1)-Asp 206(OD1), Arg 492(NE)-Asp 206(OD1), Arg 492(N1)-Asp 206(OD2) and Tyr 497(NE)-Asp 210(OD1). A series of new compounds was gained through searching the fragment database derived from ZINC based on the known inhibitor-compound 7 by the means of “replace fragment” technique. The compounds acquired via meeting the requirements of the absorption, distribution, metabolism, and excretion (ADME) predictions. Finally, 12 compounds with better binding affinity were identified. The comp#1, as a representative, was selected to be synthesized and assayed for their CDC25B inhibitory activities. The comp#1 exhibited mild inhibitory activities against human CDC25B with IC_50_ values at about 39.02 μM. Molecular Dynamic (MD) simulation revealed that the new inhibitor-comp#1 had favorable conformations for binding to CDC25B and disturbing the interactions between CDC25B and CDK2/Cyclin A.

## INTRODUCTION

According to the latest statistics of morbidity and mortality in cancer, more than 8.2 million people died and approximately 14 million new cases appeared in 2012, more seriously, the deaths will rise to 13 million annually and an estimated 22 million new cases will arise per year over the next two decades [1]. Cancers of lung (19.4% of the total), liver (9.1%), and stomach (8.8%) processed the highest mortality, which are caused by disordered cell cycle and irregularities such as deletions, over-expression, or mutations in the molecules [2–4]. Therefore, the urgent need for identifying novel and efficient agents to cure the cancer should be paid more attentions.

Cell division cycle 25 (CDC25) dual specificity phosphatases which consist of CDC25A, CDC25B and CDC25C acted as important regulators of cell cycle progression by activating the cyclin-dependent kinases (CDKs) through removal of inhibitory phosphates from tyrosine and threonine residues [5, 6]. The cell cycle is divided into four phases: a Gap phase (G1), the phase of DNA replication (S), a second Gap phase (G2) and mitosis (M), which splits itself into two daughter cells. CDC25B plays a significant role in regulating the G2/M phase transition through the activity on CDK2 kinase [7, 8]. In view of the role in cell cycle progression, it is apparently that CDC25B is closely related with oncogenesis. The over-expression of CDC25B is often found in numerous human tumors, including lung cancer cells [9], liver tumor cells [10] and stomach cancer cells [11], resulting in an excess of CDK2/Cyclin A activation and accelerating cell proliferation, which is associated with a poor clinical prognosis. Therefore, CDC25B has become an attractive anticancer target [12–14].

In fact, quite a lot of efforts on discovering CDC25B phosphatase inhibitors have been conducted for many years. However, most potent inhibitors, including quinonoids, phosphate surrogates and electrophilic entities, might irreversibly oxidize the catalytic cysteine in the active site of CDC25B enzyme which are conserved among many PTPs [15, 16] or cause the production of reactive oxygen species (ROS) enhancing their potential toxicity and limiting their applications in therapy [17]. Indeed, there are two obstacles on the way to find new potent inhibitors of CDC25B. Firstly, due to the crystal structure of the catalytic domains of CDC25B [18] with a small and shallow active site, it's not easy to provide a well-defined binding pocket. Secondly, the highly reactive catalytic cysteine in the active site of CDC25B hampers discovering CDC25B inhibitors [15]. Thus, one therapeutic strategy focuses on identifying potential compounds which are able to disrupt transient interactions between CDC25B and its CDK2/Cyclin A substrate.

Many studies have indicated that computer aided drug design (CADD) such as molecular docking [19], protein-protein docking [20, 21] and molecular dynamic simulation [22, 23], has been a time-saving and cost-reduction approach to find novel and efficient agents.

The compound 7 with the sulfate moiety attached to the 2-fluoro-4-hydroxybenzonitrile core [24] can disrupt the protein-protein interaction of CDC25B with CDK2/Cyclin A substrate and inhibit the phosphatase activity of CDC25B with weak inhibition(IC_50_ 1 mM). It is a very good starting point for the development of more potent inhibitors through disrupting the protein-protein interactions of CDC25B with CDK2/Cyclin A substrate based on the compound 7.

The present study was initiated in an attempt to screen the fragment database hoping to find new CDC25B inhibitors for treating cancer with the help of Discovery Studio 3.5 software (DS 3.5; Accelrys Co. Ltd, San Diego, California, USA). Meanwhile, the techniques of ZDOCK and RDOCK were used to construct the most optimized 3D structure of CDK2/Cyclin A in complex with CDC25B, followed by “replace fragment” to modify the lead compound-compound 7. The ADME predictions were also used to evaluate whether the new inhibitors found here possessed great potential to become promising drug candidates. The comp#1, as a representative, was selected to be synthesized and assayed for their CDC25B inhibitory activities. The comp#1 exhibited mild inhibitory activities against human CDC25B with IC_50_ values at about 39.02 μM. Subsequently, CDOCKER and MD simulation were utilized to analyze the binding interactions between the potential inhibitor and receptor. Molecular mechanics/Poisson-Boltzmann surface area (MM/PBSA) was further employed to explore the relationships between the inhibitor and receptor.

## RESULTS AND DISCUSSION

### Acquisition of the structure of CDC25B:CDK2/Cyclin A complex

Since no CDC25B:CDK2/Cyclin A complex has been reported, ZDOCK and RDOCK programs were used to obtain the complex structure of CDC25B: CDK2/Cyclin A. ZDOCK is a docking program that predicts all possible binding poses in the translational and rotational space between the ligand and receptor and evaluates each pose using ZDOCK score, an energy-based scoring function [28]. ZDOCK generated 60 clusters containing 2000 structures and ranked them according to their ZDOCK score. While, RDOCK is a program designed to refine and re-rank top predictions from ZDOCK using energy minimization algorithm [26]. The top 100 poses selected from ZDOCK were further refined and evaluated by E_RDOCK. The results derived from RDOCK involving five CDC25B:CDK2/Cyclin A binding modes have been listed in Table 1.

**Table 1:** ZDOCK and RDOCK score of CDC25B:CDK2/Cyclin A complex

Receptor protein	Ligand protein	Pose NO.	ZDOCK score^a^	E_RDOCK Score^a^	Clash^b^
CDC25B	CDK2/Cyclin A	1	16.42	−24.38	0
		2	13.56	−23.06	0
		3	14.30	−19.06	0
		4	13.46	−17.97	0
		5	14.18	−16.83	0

^a^Lower values of E_RDOCK and higher ZDOCK score indicate top/better docking of the complex.

^b^Clash ‘0′ indicates no stearic clash between the proteins after refinement by RDOCK protocol.

**Figure 1:**
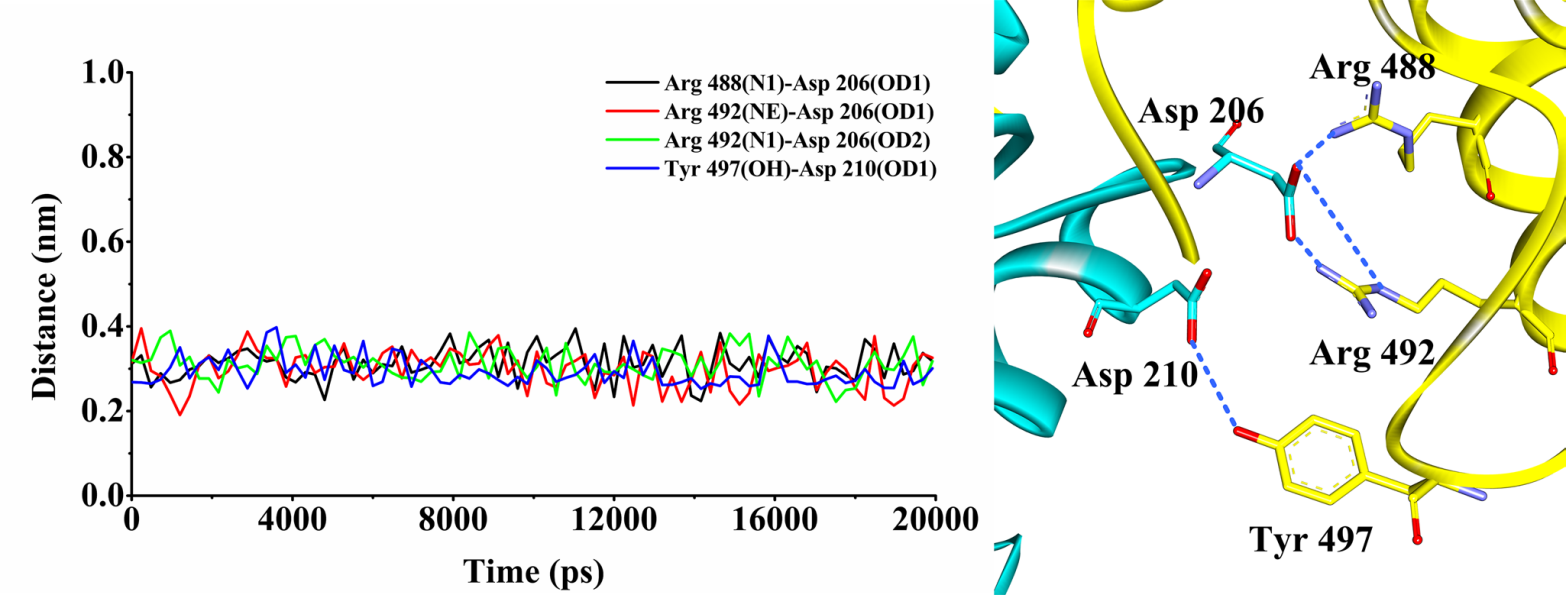
The distance variations of the four hydrogen bonds during MD simulation The black line indicated the distance variation for Arg 488(N1)-Asp 206(OD1); the red line indicated the distance variations for Arg 492(NE)-Asp 206(OD1); the green line indicated the distance variations for Arg 492(N1)-Asp 206(OD2); and the blue line indicated the distance variation for Tyr 497(OH)-Asp 210(OD1). The CDC25B protein is shown in yellow color solid ribbon while CDK2/Cyclin A protein is in cyan. The blue dotted lines indicated the H-bond interactions between the residues of the CDC25B and the residues of CDK2/Cyclin A.

To evaluate whether the docking protocol was reliable, the validation was performed using the method mentioned above. The RMSD between the five docked poses and their actual X-ray poses in the crystal structure was 0.32 Å, 0.40 Å, 0.35 Å, 0.44 Å, 0.36 Å for CDC25B and 0.40 Å, 0.38 Å, 0.42 Å, 0.34 Å, 0.43 Å for CDK2/Cyclin A, respectively, indicating that these docked poses could be used to further validate by MD simulations. Arg 488, Arg 492 and Tyr 497 in CDC25B have been served as crucial residues in substrate recognition which were found to contact with Asp 206 and Asp 210 in CDK2/Cyclin A. The distances of 4 hydrogen bonds (Arg 488(N1)-Asp 206(OD1), Arg 492(NE)-Asp 206(OD1), Arg 492(N1)-Asp 206(OD2) and Tyr 497(OH)-Asp 210(OD1)) of the five binding modes were monitored and calculated. The mean distances of the four hydrogen bonds (Arg 488(N1)-Asp 206(OD1), Arg 492(NE)-Asp 206(OD1), Arg 492(N1)-Asp 206(OD2) and Tyr 497(OH)-Asp 210(OD1)) (pose 1) maintained at 0.31 nm, 0.30 nm, 0.31 nm and 0.29 nm, respectively (Figure 1). This means that each distance was within the scope of the reported distance [31, 32]. However, the mean distances of the other four poses were moved out of the reasonable distances, indicating that pose 1 was reliable to further analysis.

### Acquisition of CDC25B Inhibitors

**Figure 2:**
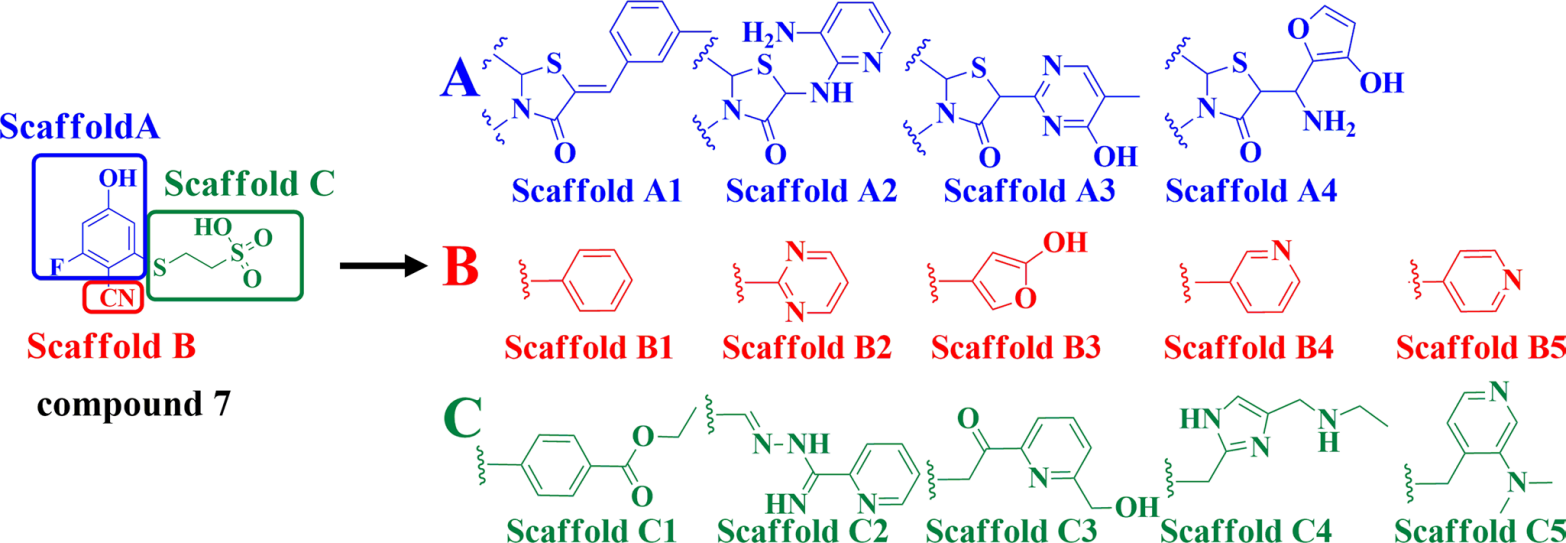
Illustration to show how to generate the 100 derivative compounds from compound 7

The detailed process of “replace fragment” for discovering the desired inhibitors is shown in Figure 2. The structure of compound 7, which is regarded as a lead compound- CDC25B inhibitor to develop novel therapeutic agents for treating cancer with an IC_50_ of 1 mM, was divided into three parts, Scaffold A, Scaffold B and Scaffold C (Figure 2). By the means of “replace fragment” technique, the three parts were replaced through searching the fragment database constructed by “Generate fragment libraries” which were mentioned in materials and methods. The 1st step was aimed at the A part generating 4 scaffolds, Scaffold A1, Scaffold A2, Scaffold A3, Scaffold A4. The 2nd step was aimed at the B part generating 5 scaffolds, Scaffold B1, Scaffold B2, Scaffold B3, Scaffold B4 and Scaffold B5 to replace B. The 3rd step was aimed at C part generating 5 scaffolds, Scaffold C1, Scaffold C2, Scaffold C3, Scaffold C4 and Scaffold C5, to replace C. Hence, there were approximately 100 different combinations thus generated. Subsequently, all the compounds were then screened by meeting the requirements of ADME to exclude non drug like compounds, finally, 24 compounds were obtained.

Before docking, the binding pocket of CDC25B:CDK2/Cyclin A was defined by a set of key residues of the receptor with a distance less than 5 Å from a heavy atom of the ligand. To evaluate the applicability of CDOCKER in the present study, the original co-crystal ligand-compound 7 from the PDB structure was re-docked into the X-ray structure of the receptor. The root mean square deviation (RMSD) between the docked poses and their actual X-ray poses in the crystal structure are 1.1955 Å for CDC25B:CDK2/Cyclin A complex [29]. Hence, the 24 hits were further docked into the binding pocket of CDC25B:CDK2/Cyclin A using the method we have described earlier. The compound 7, as a positive control compound, was selected to compare the docking results of other compounds. The top 12 compounds which showed higher binding energy than compound 7(-CDOCKER_ENERGY= 18.46 kcal/mol) were gained and listed in Table 2. Of the 12 derivatives, the comp#1, has the strongest binding affinity with CDC25B:CDK2/Cyclin A, and hence it is singled out for further investigation.

**Table 2:** The compound 7 was used as a positive control, and the 12 compounds (comp#1-#12) were ranked roughly according to the strength of their docking scores to the receptors

compound	Combination of Scaffolds^a^	-CDOCKER(Kcal/ mol)	ADME properties predicted			
			Aqueous solubility_Level^b^	Human intestinal absorption (HIA)_Level^c^	ADME_EXT_PPB# Prediction^d^	ADME_EXT_CYP2D6#Prediction^e^
compound 7	A-B-C	18.46	4	0	FALSE	FALSE
comp#1	A1-B1-C1	32.39	2	1	TRUE	FALSE
comp#2	A2-B1-C1	32.31	2	0	TRUE	FALSE
comp#3	A3-B1-C1	31.98	2	0	TRUE	FALSE
comp#4	A4-B1-C1	30.91	2	0	TRUE	FALSE
comp#5	A1-B2-C1	29.91	2	0	TRUE	FALSE
comp#6	A1-B3-C1	29.69	2	0	TRUE	FALSE
comp#7	A1-B4-C1	29.51	2	0	TRUE	FALSE
comp#8	A1-B5-C1	27.39	2	0	TRUE	FALSE
comp#9	A1-B1-C2	27.19	2	0	TRUE	FALSE
comp#10	A1-B1-C3	26.43	2	0	TRUE	FALSE
comp#11	A1-B1-C4	25.21	2	0	TRUE	FALSE
comp#12	A1-B1-C5	25.01	2	0	TRUE	FALSE

^a^See Figure 2 for the structure of scaffolds.

^b^Aqueous solubility: solubility levels were divided into 0-5 which meant extremely low; no, very low, but possible; yes, low; yes, good; yes, optimal; no, too soluble, respectively.

^c^HIA: HIA levels of 0-3 reflected good, moderate, low, and very low absorption levels, respectively.

^d^CYP2D6 binding activity: CYP2D6 was included in the metabolism of different drugs in the liver. For loss of function of the enzyme, CYP2D6 inhibitors would rise the concentration of a drug. False and True meant non-inhibitor and inhibitor.

^e^PPB: True and False reflected on binding as < 90% (good bioavailability), binding as > 90%.

**Figure 3:**
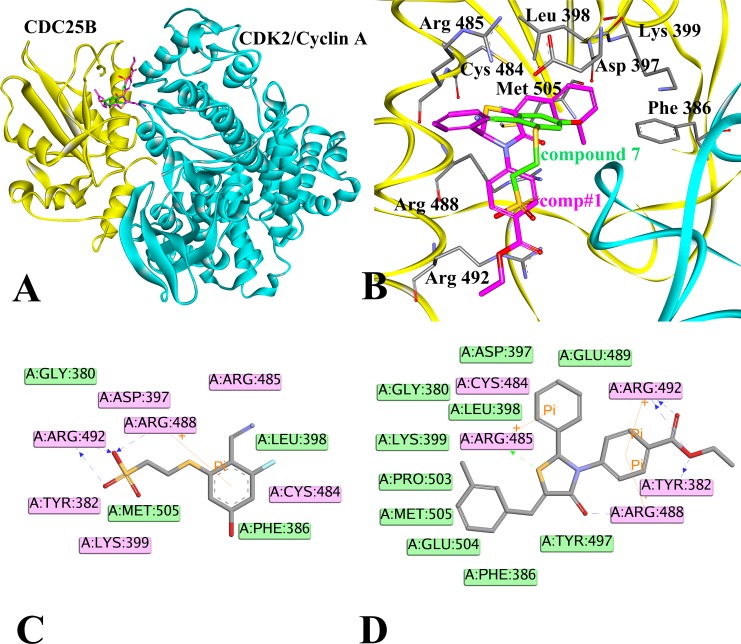
Illustration to show the conformations obtained by docking compound 7 and comp#1, respectively, to CDC25B (**A**) The compound 7 (green) and comp#1 (magenta) bound to CDC25B:CDK2/Cyclin A. (**B**) Interactions of compound 7 (green) and comp#1 (magenta) with active site amino acids of CDC25B protein. (**C**) The ligand-protein interaction diagram of compound 7 with CDC25B (PDB ID: 4WH9). (**D**) The ligand-protein interaction diagram of comp#1 with CDC25B (PDB ID: 4WH9). The CDC25B protein was shown in yellow color solid ribbon while CDK2/Cyclin A protein was in cyan. The blue and green dotted lines indicated the H-bond interactions of the receptor with compound 7 and comp#1, respectively. Pi interactions were represented by an orange line. Residues involved in hydrogen-bond, charge or polar interactions were represented by pink rectangles. Residues involved in van der Waals interactions were represented by green rectangles.

The results of receptor-ligand interactions obtained from the docking simulation were shown in Figure 3, where panel A displayed the binding pattern of the reference inhibitor compound 7 and comp#1 in the active site of CDC25B:CDK2/Cyclin A, panel B described the interactions between CDC25B and compound 7 (carbon atoms colored in green) and comp#1 (carbon atoms colored in magenta) in the active site (Phe386, D397, Leu398, K399, Cys484, R485, Arg488, Arg 492 and Met505), while panel C and D showed the 2D diagrams between CDC25B and compound 7 and comp#1, respectively.

The pink rectangles in 2D diagram mainly represented polar interactions including hydrogen bonds and charge interactions and the green rectangles represented nonpolar interactions including van der waals interactions and hydrophobic interactions. In the 2D diagram of CDC25B-compound 7, shown in Figure 3C, compound 7 could form van der waals and hydrophobic interactions with the residues such as Gly 380, Phe 386, Leu 398, Leu 500 and hydrogen bonds and charge interactions with the residues such as Tyr 382, Asp 397, Lys 399, Cys 484, Arg 485, Arg 488 and Arg 492. The phenyl ring of part A in compound 7 inserted into the pocket formed by the side chains of Leu 398 and Arg 488, forming hydrophobic and cation-Pi interactions, respectively. The nitrile nitrogen of part B and fluorine of part A in compound 7 pointed towards the backbone of Arg 485 and Cys 484, respectively. The three conservative H-bond interactions formed between O1,O3 of part C in compound 7and the two key residues of CDC25B, Arg 488 and Arg 492 required for CDK2 substrate recognition [32] was observed in our docking study. The Figure 3D described the 2D diagram of CDC25B-comp#1, showing that hydrogen bond, charge interactions were formed by the residues such as Tyr 382, Cys 484, Arg 485, Arg 488 and Arg 492, while van der waals and hydrophobic interactions could be generated with the residues such as Phe 386, Asp 397, Leu 398, Lys 399, Glu 489, Tyr 497, Pro 503, Glu 504 and Met 505. The Scaffold A1 in comp#1 (Figure 2) located in the same place to part A in compound 7, buried into the hydrophobic area, including Phe 386, Leu398, Lys 399, Pro503, Glu 504 and Pro 505. The sulphur of Scaffold A1 in comp#1 oriented to Arg 485, forming a hydrogen bond. The O9 of Scaffold A1 and O27, O28 of Scaffold C1 of comp#1 formed four hydrogen bonds with the residues of Tyr 382, Arg 488 and Arg 492, respectively. The phenyl ring of Scaffold C1 in comp#1 formed two cation-Pi interactions with the residues of Arg 492 and Arg 488. In addition, the Scaffold B1 also formed a cation-Pi interaction with residue Arg 485. In conclusion, comp#1 not only formed two more hydrogen bonds and two more cation-Pi interactions with the receptor CDC25B than compound 7, accounting for the higher docking score of comp#1 to CDC25B than compound 7.

### Chemistry

**Scheme 1:**
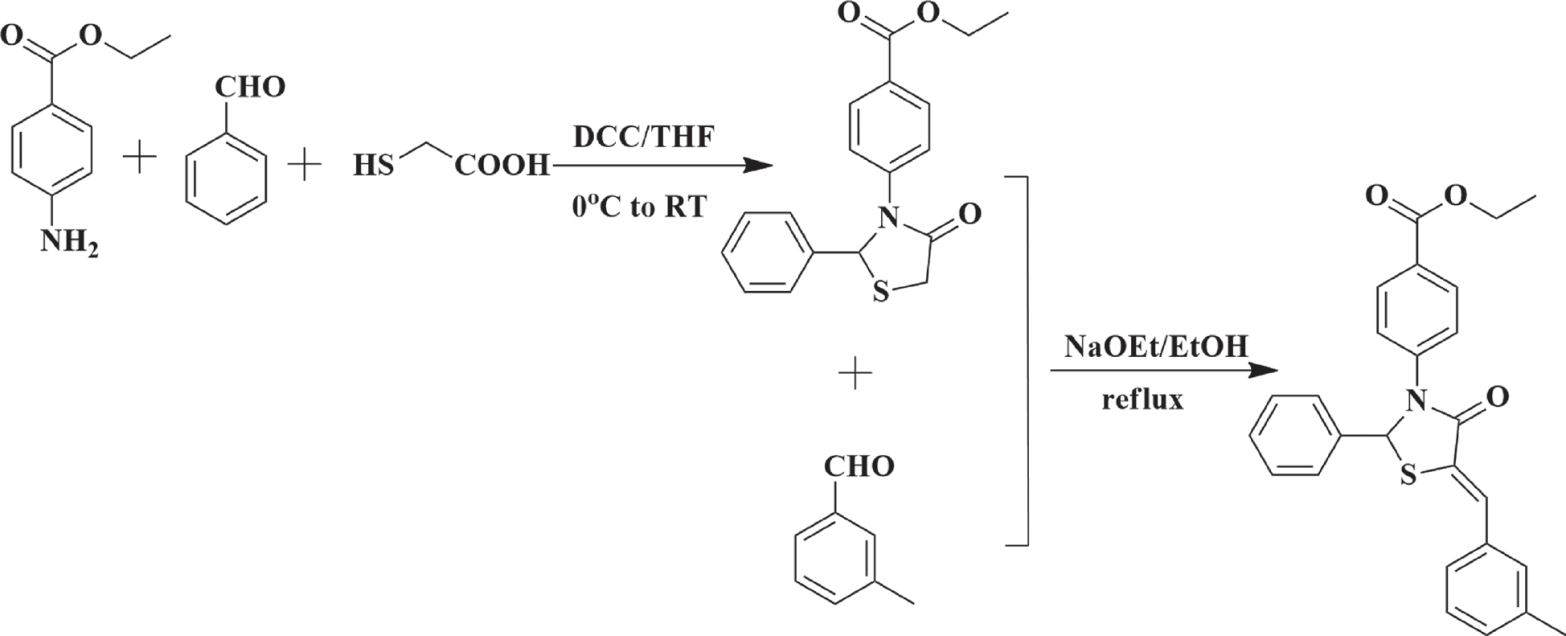
Reaction scheme for synthesis of comp#1

In the present work, we just chose comp#1 as a representive compound to synthesize. The main purpose here is to test the correctness of the hypothesis. The comp#1 was synthesized as shown in Scheme 1.

Ethyl 4-(4-oxo-2-phenylthiazolidin-3-yl)benzoate

To a solution of methyl triphenyl phosphonium bromide (8.26 g, 50 mmol) in dry tetrahydrofuran (THF) (300 mL) was added benzaldehyde (10.61 g, 100 mmol) at 0°C and the reaction stirred for 5 min. Then 2-mercaptoacetic acid (7.00 mL, 100 mmol) was added dropwise and DCC (12.40 g, 60 mmol) was added in batches and the reaction was stirred at 0°C for 5 min. Then, the reaction mixture was naturally heated to room temperature and stirred at room temperature for 3 h. TLC and LC-MS examination showed that most of the starting material was converted into the target compound. The mixture was extracted with ethyl acetate (350 mL) (×3), and washed with 5% citric acid (70 ml) (×3), water (70 ml) (×3), 5% NaHCO_3_ (70 ml) (×3) and saturated brines (70 ml) (×3), dried over MgSO_4_ and filtered and concentrated in vacuo to give the crude product as light yellow oil. Purification by column chromn chromatography (200–300 mesh silica gel, PE: ethyl acetate=2.5:1) gave final product (13.07 g, 80%).^1^H-NMR (DMSO-*d*_6_, 400MHz): δ 7.83–7.86 (2H, m, Ar-H); 7.49–7.51 (2H, q, *J*=8.8Hz, Ar-H); 7.36–7.38 (2H, t, *J*=8.4Hz, Ar-H); 7.19–7.25 (3H, m, Ar-H); 6.63(1H, s, CH); 4.24 (2H, q, *J*=7.2Hz, CH_2_); 3.89–4.06 (2H, m, CH_2_); 1.26 (3H, t, *J*=7.2Hz, CH_3_); MS (*m/z*): 328.2(M+1); 350.2 (M+Na); 677.1(2M+Na).

(Z)-ethyl 4-(5-(3-methylbenzylidene)-4-oxo-2-phenylthiazolidin-3-yl)benzoate (comp#1).

To a well stirred solution of the ethyl 4-(4-oxo-2-phenylthiazolidin-3-yl)benzoate (4.1 g, 4 mmol) in dry EtOH (30 mL) was added 3-methylbenzaldehyde (65 g, 4 mmol), NaOEt and the reaction was stirred at room temperature for 0.5 h. TLC and LC-MS examination showed that most of the starting material was converted into the target compound. The precipitated product was filtered, and purified by recrystallization from dry EtOH to yield comp#1 (1.45 g, 85%).^1^H-NMR(DMSO-*d*_6_, 400MHz): δ 7.19–7.91 (13H, m, Ar-H); 7.55 (1H, s, =CH); 7.02 (1H, s, CH); 4.259 (2H, q, *J*=7.2 Hz, OCH_2_); 1.27 (3H, t, *J*=7.2 Hz, CH_3_); MS (*m/z*): 428.3(M-1); 452.2(M+Na).

The inhibitor influencing the interaction between CDC25B and CDK2/Cyclin A.

The 20 ns Molecular dynamics were performed to characterize the internal motions of the inhibitor-complexed system including CDC25B:CDK2/Cyclin A-comp#1, CDC25B:CDK2/Cyclin A-compound 7 and the inhibitor-uncomplexed system CDC25B:CDK2/Cyclin A complex.

**Figure 4:**
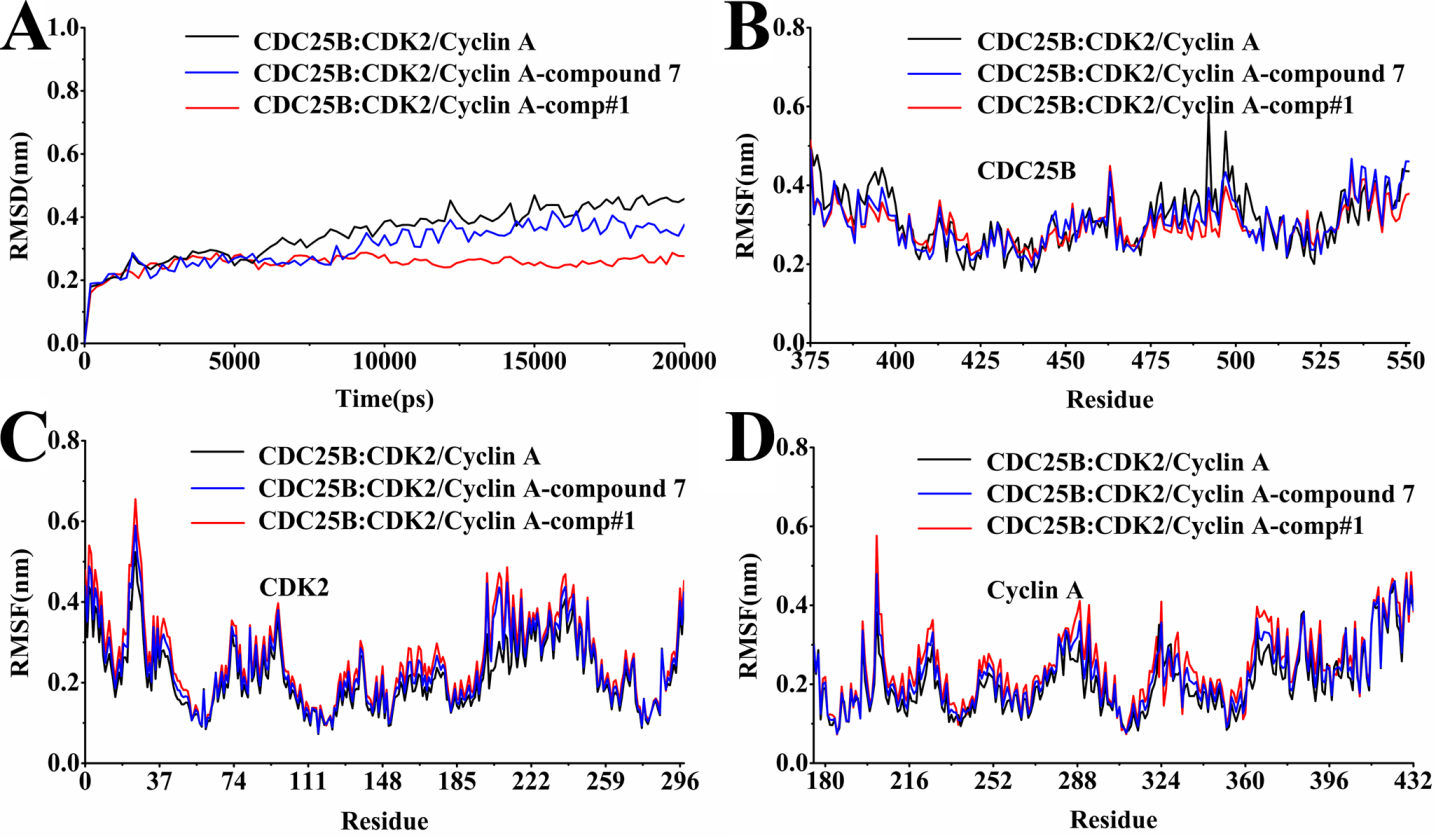
Analysis of molecular dynamics simulations (**A**) The RMSDof all backbone atoms for the receptor CDC25B:CDK2/Cyclin A. (**B**) The RMSF of the side-chain atoms for the receptor CDC25B. (**C**) The RMSF of the side-chain atoms for the receptor CDK2. (**D**) The RMSF of the side-chain atoms for the receptor Cyclin A. The black line indicated the outcome for the system of the receptor alone without any ligand, the blue line for that of the receptor with the ligand compound 7, and the red line for that of the receptor with the ligand comp#1.

The RMSD is an evaluative criterion used to estimate the stability of a protein and protein-ligand systems. Displayed in the Figure 4A was the backbone RMSD curves for CDC25B:CDK2/Cyclin A without ligand and their complexes with comp#1 and compound 7. The RMSD of CDC25B:CDK2/Cyclin A-comp#1, CDC25B:CDK2/Cyclin A-compound 7 and CDC25B:CDK2/Cyclin A-uncomplexed system was shown in Figure 4A and all of the characters concerned reached stable at nearly 2 ns. The RMSD curve of CDC25B:CDK2/Cyclin A-comp#1 is more stable than CDC25B:CDK2/Cyclin A -compound 7 and CDC25B:CDK2/Cyclin A without ligand.

Furthermore, the root mean square fluctuations (RMSF) for all the side-chain atoms of the receptors was also calculated for in depth analysis of the interactions of the three complexes, CDC25B:CDK2/Cyclin A-comp#1, CDC25B:CDK2/Cyclin A-compound 7 and CDC25B:CDK2/Cyclin A-uncomplexed system. It can be seen from the Figure 4B–4D that comp#1 and compound 7 both bound to CDC25B:CDK2/Cyclin A altered the RMSF of the key amino acid residues, respectively. The RMSF fluctuated large, which meant the residues were unstable, whereas the RMSF fluctuated small, which meant the residues were stable.

The plot B displayed the residues of CDC25B in CDC25B:CDK2/Cyclin A-comp#1 and CDC25B:CDK2/Cyclin A-compound 7 complexes shared similar RMSF distributions and similar trends of dynamic features. The three regions (Gly 380-Phe 386, Thr 390-Tyr 400, Cys 484-Tyr 506) fluctuated lower in CDC25B:CDK2/Cyclin A-comp#1 system than CDC25B:CDK2/Cyclin A-compound 7 system, which means the inhibitor comp#1 made CDC25B more stable than compound 7 did. The Figure 4C and 4D showed the fluctuation of RMSF in CDK2/Cyclin A after the inhibitor comp#1 and compound 7 bound to CDC25B:CDK2/Cyclin A. It is noteworthy that almost all residues in CDK2/Cyclin A displayed minor difference in the overall curves, except the residues Asp 206-Asp 210 in CDK2 of CDC25B CDC25B:CDK2/Cyclin A-comp#1 and CDC25B:CDK2/Cyclin A-compound 7. Asp 206 and Asp 210 played an important role in binding to CDC25B. Thus, it can be concluded that the inhibitors comp#1 and compound 7 both bound to CDC25B reduced the contact between the protein CDC25B and CDK2/Cyclin A.

To understand the specific interactions between protein-protein and protein-ligand complexes, the binding free energy was calculated using MM/PBSA approach composed of four terms, i.e., the van der waals interaction energy, the electrostatic energy, the polar solvation free energy, and the non-polar solvation free energy. For all systems, each snapshot structures were extracted during equilibrium phase in the last 10 ns trajectory to calculate the binding free energy.

The binding free energy and detailed contributions of the four energy components obtained from the MM/PBSA calculation of the protein-protein and protein-ligand complexes are listed in Table 3. As shown in Table 3, the binding energy of the CDC25B:CDK2/Cyclin A was the lowest among the three protein-protein complexes, i.e., CDC25B:CDK2/Cyclin A (binding with comp#1), CDC25B:CDK2/Cyclin A (binding with compound 7) and CDC25B:CDK2/Cyclin A. The binding energy of CDC25B:CDK2/Cyclin A was estimated to be -171.806 kJ/mol, and the CDC25B:CDK2/Cyclin A (binding with comp#1) and CDC25B:CDK2/Cyclin A (binding with compound 7) were respectively estimated to be -3.638 kJ/mol and -42.449 kJ/mol. We can clearly see that the electrostatic interactions were the major favorable contributions to the binding energies between CDK2/Cyclin A and CDC25B in the three systems. However, the contributions of electrostatic interactions negatively contributed to the total energy were completely counteracted by the opposite stronger polar solvation. Therefore, taking van der waals, electrostatic interaction, non-polar solvation and polar solvation into considerations, CDC25B:CDK2/Cyclin A was the most stable system, which meant that the ligands comp#1 and compound 7 both decreased the binding affinity between CDK2/Cyclin A and CDC25B.

**Table 3:** Binding free energies (kJ/mol) and its components between protein and protein, protein and ligand, respectively

complex	Van der Waal (kJ/mol)	Electrostatic (kJ/mol)	Polar solvation (kJ/mol)	Non-polar solvation (kJ/mol)	Binding energy (kJ/mol)
CDC25B:CDK2/Cyclin A^a^	−725.772	−1146.987	1778.599	−77.645	−171.806
CDC25B:CDK2/Cyclin A (binding with comp#1)^b^	−844.393	−1164.718	2097.316	−91.844	−3.638
CDC25B:CDK2/Cyclin A (binding with compound 7)^c^	−870.918	−1415.664	2333.299	−89.165	−42.449
CDC25B:CDK2/Cyclin A-comp#1^d^	−311.415	−11.098	127.710	−21.527	−216.329
CDC25B:CDK2/Cyclin A-compound 7^e^	−121.681	−445.412	538.187	−13.864	−42.770

^a^CDC25B:CDK2/Cyclin A: the binding free energy between CDC25B and CDK2/Cyclin A.

^b^CDC25B:CDK2/Cyclin A (binding with comp#1): the binding free energy between CDC25B and CDK2/Cyclin A when comp#1 bound to CDC25B.

^c^CDC25B:CDK2/Cyclin A (binding with compound 7): the binding free energy between CDC25B and CDK2/Cyclin A when compound7 bound to CDC25B:CDK2/Cyclin A.

^d^CDC25B:CDK2/Cyclin A-comp#1: the binding free energy between comp#1 and CDC25B:CDK2/Cyclin A.

^e^CDC25B:CDK2/Cyclin A-compound 7: the binding free energy between compound 7 and CDC25B:CDK2/Cyclin A.

To Figure out how the ligands influenced the interactions between CDC25B and CDK2/Cyclin A, the binding free energy between the protein and ligand were also calculated. The Table 3 displayed that CDC25B:CDK2/Cyclin A-comp#1 possessed higher negative binding energy value of -216.329 kJ/mol than CDC25B:CDK2/Cyclin A-compound 7 complex with value of -42.770, indicating that the system of CDC25B:CDK2/Cyclin A - comp#1 seems more stable than CDC25B:CDK2/Cyclin A-compound 7. Van der waals, electrostatic interactions and non-polar solvation energy made a negative contribution to the total interaction energy while only polar solvation energy made a positive contribution to the total free binding energy, indicating that van der waals, electrostatic interactions and non-polar solvation energy together are in favor of the stability of the CDC25B:CDK2/Cyclin A-comp#1 and CDC25B:CDK2/Cyclin A-compound 7 complexes. For negative contribution, van der waals interactions offered greater contributions than electrostatic interactions and the non-polar free energy which possessed less the total binding energy as for the CDC25B:CDK2/Cyclin A-comp#1 system, while for CDC25B:CDK2/Cyclin A-compound 7 system, electrostatic interactions possessed the most among the three components. Consistent with the results of molecular docking (Figure 3), van der waals interactions played the main role in CDC25B:CDK2/Cyclin A-comp#1 system, while in CDC25B:CDK2/Cyclin A -compound 7 system, electrostatic interactions played the major role. In addition, compared with CDC25B:CDK2/Cyclin A-compound 7 system, comp#1 bound to CDC25B:CDK2/Cyclin A exhibited higher van der waals, representing much more hydrophobic interactions, which promoted the stability of CDC25B:CDK2/Cyclin A-comp#1 system.

Hence, due to the ligand bound to CDC25B, it changed the conformation of the protein CDC25B, thus, decreased the binding energy between CDC25B and CDK2/Cyclin A. In addition, the system of CDC25B:CDK2/Cyclin A-comp#1 seemed more stable than CDC25B:CDK2/Cyclin A-compound 7, accounting for the high energy between CDC25B and CDK2/Cyclin A when bound to comp#1.

**Figure 5:**
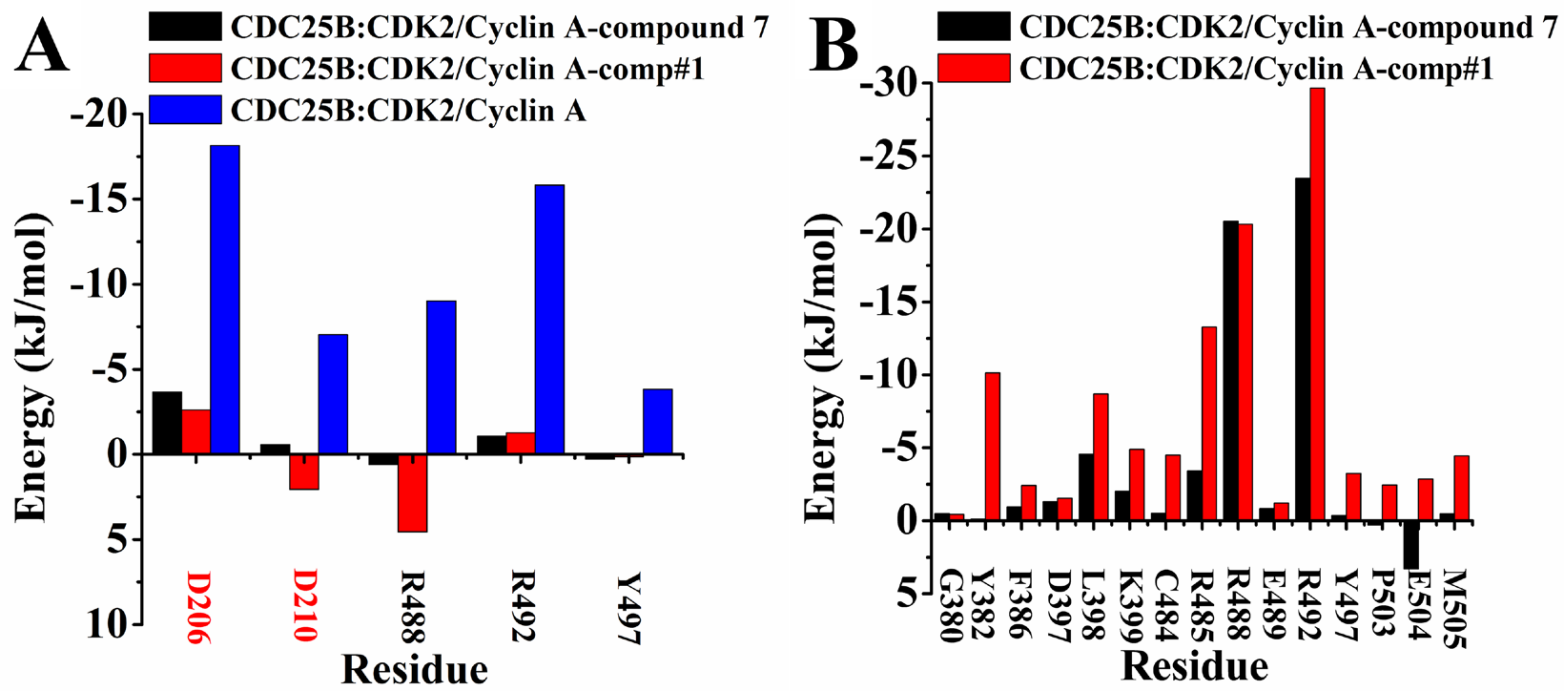
Decomposition of binding energy on a per-residue basis, only residues making significant favorable contribution were shown (**A**) Interaction energies between CDC25B and CDK2/Cyclin A. The residues of CDC25B were marked in black, while the residues of CDK2/Cyclin A were marked in red. (**B**) Interaction energies between CDC25B and comp#1 (red) and compound 7 (black), respectively. Black for the system of CDC25B:CDK2/Cyclin A-compound 7, red for CDC25B:CDK2/Cyclin A-comp#1 and blue for CDC25B:CDK2/Cyclin A.

The binding energy decomposition method by residues was employed to better understand how comp#1 and compound 7 influenced the interactions between CDC25B and CDK2/Cyclin A. The Figure 5 displayed the contributions of individual residue to the whole binding for CDC25B:CDK2/Cyclin A (binding with comp#1), CDC25B:CDK2/Cyclin A (binding with compound 7) and CDC25B:CDK2/Cyclin A systems. It is observed that the residues (Arg 488, Aeg 492 and Tyr 497) in CDK interactions site of CDC25B and Asp 206, Asp 210 of CDK2/Cyclin A displayed large conformational change. Previous study has demonstrated that the residue Asp 206 of CDK2/Cyclin A formed three hydrogen bonds with Arg 488 and Arg 492 of CDC25B, and residue Asp 210 of CDK2/Cyclin A formed a hydrogen bond with Tyr 497 of CDC25B [32]. Compared with the system of CDC25B:CDK2/Cyclin A, the energy of five important residues showed an obvious increase, the decomposition energy of Arg 488, Arg 492 and Tyr 497 in CDC25B (bind with comp#1) varies in the range from -9.01 kJ/mol to 4.55 kJ/mol, -15.83 kJ/mol to -1.27 kJ/mol and -3.82 kJ/mol to 0.14 kJ/mol, respectively, and in CDC25B (binding with compound 7) were from -9.01 kJ/mol to 0.59 kJ/mol, -15.83 kJ/mol to -1.07 kJ/mol and -3.82 kJ/mol to 0.28 kJ/mol, respectively, while the decomposition energy of Asp 206 and Asp 210 in CDK2/Cyclin A (binding with comp#1) was from -18.15 kJ/mol to -2.61 kJ/mol and -7.04 kJ/mol to 2.06 kJ/mol, respectively, and in CDK2/Cyclin A (binding with compound 7) changed from -18.15 kJ/mol to -3.65 kJ/mol and -7.04 kJ/mol to -0.57 kJ/mol, respectively, (Figure 5A). The rise of the binding energy declared that the interactions between the two proteins have been weakened. The comp#1 and compound 7 occupied the position of CDK interaction site in CDC25B, which caused the decrease the contacts between the key residues, accounting for the increase of the decomposition energy.

A further analysis between protein-ligand systems, including CDC25B:CDK2/Cyclin A-comp#1 and CDC25B:CDK2/Cyclin A-compound 7 complexes was shown in Figure 5B to better understand how comp#1 and compound 7 were bound to CDC25B:CDK2/Cyclin A, respectively. The key residues were observed in three significantly different regions (Gly 380-Phe 386, Thr 390-Tyr 400, Cys 484-Tyr 506) of CDC25B, which was in accordance with the RMSF results in Figure 4. The major residues of CDC25B in CDC25B:CDK2/Cyclin A-comp#1 and CDC25B:CDK2/Cyclin A-compound 7 contributed to the binding free energy varied in the range from -0.43 to -22.66 kJ/mol and -23.48 to 3.31 kJ/mol, respectively. For CDC25B:CDK2/Cyclin A-compound 7, the key residues of CDC25B were Gly 380, Tyr 382, Phe 386, Asp 397, Leu 398, Lys 399, Cys 484, Arg 485, Arg 488, Arg 492, Met 505, while some other residues (Glu 489, Tyr 497, Pro 503, Glu 504) also played an important role in CDC25B:CDK2/Cyclin A-comp#1 system. Compared with compound 7, the energy of amino acids Tyr 382, Leu 398, Cys 484, Arg 485 and Met 505 of CDC25B:CDK2/Cyclin A-comp#1 system showed remarkable decrease. The decomposition energy changed from -0.13 kJ/mol to -10.14 kJ/mol, -4.56 kJ/mol to -8.69 kJ/mol, -0.51 kJ/mol to -4.49 kJ/mol, -3.42 kJ/mol to -13.27 kJ/mol and -0.47 kJ/mol to -4.45 kJ/mol, respectively. In addition, the decomposition energy of residues Tyr 497, Pro 503 and Glu 504 also has a notable decrease, altering from -0.35 kJ/mol to -3.24 kJ/mol, 0.30 kJ/mol to -2.45 kJ/mol and 3.31 kJ/mol to -2.85 kJ/mol, respectively. The decrease of the binding free energy indicated that the interactions between these eight residues have been strengthened. One reason was that comp#1 bound to CDC25B increased the electronic interactions between the residues and protein CDC25B. As shown in Figure 3, the Scaffold C1 of comp#1 was pointed close to Tyr 382 and Arg 492, which was beneficial to form more stable interactions, like hydrogen bond and cation-Pi interactions. The Scaffold A1 and Scaffold B1 in comp#1 were located nearly to Arg 485, which increased the opportunity to form a hydrogen bond and cation-Pi interactions with it. The other reason was that the Scaffold A1 of comp#1 formed more hydrophobic interactions with residues Pro 503, Glu 504 and Met 505.

In summary, the decomposition energy of the residues Arg 488, Arg 492 and Tyr 497 in CDC25B and the residues Asp 206 and Asp 210 in CDK2/Cyclin A varied a lot, revealing that comp#1 and compound 7 hampered the interactions between CDC25B and CDK2/Cyclin A. Furthermore, although the binding pattern was same between comp#1 and compound 7, respectively, bound to CDC25B, comp#1 bound to CDC25B could be more stable, which made it more conducive to interrupt the contact between CDC25B and CDK2/Cyclin A.

The stability of a three-dimensional structure of the protein is decided by a subtle balance among all kinds of weak interactions, such as hydrogen bonds, conjugation interactions, hydrophobic interactions and so on, of which hydrogen bonds play the most important role in stabilizing the system.

**Figure 6:**
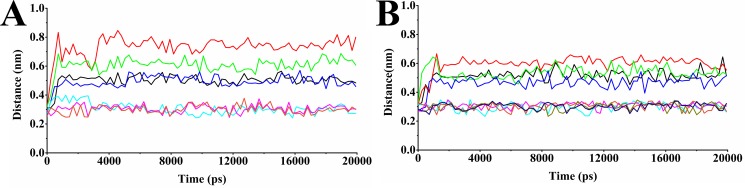
Distance graphs of some important hydrogen bonding interactions (**A**) Distances between Arg 488(N1)-Asp 206(OD1), Arg 492 (NE)-Asp 206(OD1), Arg 492(N1)-Asp 206(OD2), Tyr 497(OH)-Asp 210(OD1), Arg 488(NH1)-O1, Arg 492(NH2)-O1 and Arg 492(NE)-O3, respectively, in the system of CDC25B:CDK2/Cyclin A-compound 7. The black line indicates the distance variation for Arg 488(N1)-Asp 206(OD1); the red line indicates the distance variation for Arg 492(NE)-Asp 206(OD1); the green line indicates the distance variation for Arg 492(N1)-Asp 206(OD2); the blue line indicates the distance variation for Tyr 497(OH)-Asp 210(OD1); the cyan line indicates the distance variation for Arg 488(NH1)-O1; the magenta line indicates the distance variation for Arg 492(NH2)-O1; the orange line indicates the distance variation for Arg 492(NE)-O3. (**B**) Distances between Arg 488(N1)-Asp 206(OD1), Arg 492 (NE)-Asp 206(OD1), Arg 492(N1)-Asp 206(OD2), Tyr 382(OH)-O27, Arg 485(NH1)-S2, Arg 488(NH1)-O9, Arg 492(NH2)-O27 and Arg 492(NE)-O28, respectively, in the system of CDC25B:CDK2/Cyclin A-comp#1. The black line indicates the distance variation for Arg 488(N1)-Asp 206(OD1); the red line indicates the distance variation for Arg 492(NE)-Asp 206(OD1); the green line indicates the distance variation for Arg 492(N1)-Asp 206(OD2); the blue line indicates the distance variation for Tyr 497(OH)-Asp 210(OD1); the cyan line indicates the distance variation for Tyr 382(OH)-O27; the magenta line indicates the distance variation for Arg 485(NH1)-S2; the orange line indicates the distance variation for Arg 488(NH1)-O9; the dark yellow line indicates the distance variation for Arg 492(NH2)-O27; the navy line indicates the distance variation for Arg 492(NE)-O28.

Figure 6 displayed the distance fluctuations of the active residues with the atoms of the ligand that were included in the hydrogen bond interactions. It is observed that all the important hydrogen bonds which have been shown in the initial docking models were maintained during the MD simulations. For example, in CDC25B:CDK2/Cyclin A-compound 7 system, one hydrogen bond formed between O1 of compound 7 and Arg 488 in CDC25B and two hydrogen bonds formed between O1, O3 of compound 7and Arg 492 in CDC25B, while in CDC25B:CDK2/Cyclin A-comp#1 system, two hydrogen bonds formed between O27 of comp#1 and Tyr 382 and Tyr 492 in CDC25B, respectively, another hydrogen bond formed between S2 of comp#1 and Arg 485 and other two hydrogen bonds between O9 of comp#1 and Arg 488 and O28 of comp#1 and Arg 492, respectively, were maintained through the entire simulation.

In CDC25B:CDK2/Cyclin A-comp#1 system, the distances of five hydrogen bonds (Tyr 382-O27, Arg 485-S2, Arg 488-O9, Arg 492-O27, Arg 492-O28, respectively) were fluctuated from 0.25 nm to 0.32 nm, which remained within hydrogen bonds distance throughout the whole simulation, indicating that the five hydrogen bonds played vital role in the entire time period to stabilize the receptor-ligand complex. In CDC25B:CDK2/Cyclin A-compound 7 system, the average distances of the three hydrogen bonds (Arg 488-O1, Arg 492-O1, Arg 492-O3) were about 0.31 nm, 0.31 nm and 0.30 nm, respectively. At the beginning of the simulation, the distance of hydrogen bond interaction between Arg 488 and O1 of the compound 7 fluctuated large and was beyond the hydrogen bond distance. Then, it tended to be stable and almost remained within the reasonable hydrogen bond distance from 3 ns to the end. The other two hydrogen bonds were almost remained within hydrogen bond distance for the entire duration of the simulation. However, the hydrogen bond interactions between compound 7 and Tyr 382 and Arg 485 were absent, revealing that this hydrogen bond of comp#1 with CDC25B made the system more stable.

To Figure out whether these stable hydrogen bonds formed between the ligand (comp#1 and compound 7) and Arg 488, Arg 492 and Tyr 497 of CDC25B could influence the interactions between CDC25B and CDK2/Cyclin A, the distance between the two proteins was also calculated.

**Figure 7:**
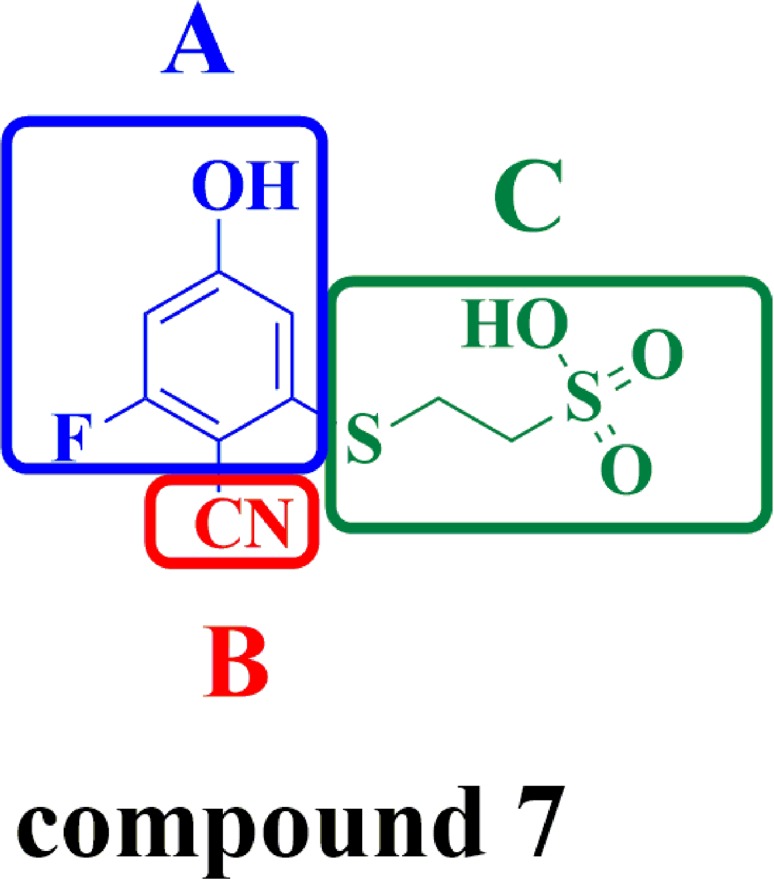
(**A**, **B** and **C**) were replaced by screening the database derived from ZINC.

As is mentioned above, the NH1 of Arg 488 and NE of Arg 492 of CDC25B formed hydrogen bonds with the OD1 of Asp 206 of CDK2/Cyclin A and the NH1 of Arg 492 formed one hydrogen bond with the OD2 of Asp 206 and the OH of Tyr 497 of CDC25B formed one hydrogen bond with the OD1of Asp 210 in CDK2/Cyclin A. The distances of the 4 hydrogen bonds (Arg 488(N1)-Asp 206(OD1), Arg 492(NE)-Asp 206(OD1), Arg 492(N1)-Asp 206(OD2) and Tyr 497(OH)-Asp 210(OD1)) was calculated. The Figure 7 and Figure 6 displayed the change of the distances between these four pair atoms in CDC25B:CDK2/Cyclin A-comp#1 system, CDC25B:CDK2/Cyclin A-compound 7 system and CDC25B:CDK2/Cyclin A system. In un-bounded system, the mean distances of the four hydrogen bonds were about 0.31 nm, 0.30 nm, 0.31 nm, 0.29 nm, respectively. When the inhibitor bound to the receptor, the distances of the corresponding atom pairs showed higher fluctuation than un-bonded system. In bounded system, the mean distances between the four atom pairs in CDC25B:CDK2/Cyclin A (binding with comp#1) were about 0.51 nm, 0.60 nm, 0.54 nm, 0.45 nm, respectively, whereas, the average distances for them in the system of CDC25B:CDK2/Cyclin A (binding with compound 7) were 0.50 nm, 0.73 nm, 0.60 nm Å, 0.49 nm, respectively. Due to the inhibitor comp#1 or compound 7 bound to CDC25B, they formed hydrogen bonds with the key residues Arg 488 and Arg 492 and altered their conformations, hence, the distances of the four atom pairs were changed and made Arg 488, Arg 492 and Tyr 497 in CDC25B far away from their paired residues Asp 206 and Asp 210 in CDK2/Cyclin A, receptively.

## CONCLUSIONS

This research focused on identifying novel and efficient inhibitors targeting CDC25B and disrupting the interactions between CDC25B and CDK2/Cyclin A by combination of computational methods including ZDOCK, RDOCK, replace fragment, ADME, molecular docking and MD simulations. The most optimized 3D structure of CDK2/Cyclin A in complex with CDC25B was constructed by the module of ZDOCK and RDOCK in Discovery Studio 3.5. Subsequently, new lead compounds with improved properties were derived using compound 7 with the help of replace fragment. Then, 12 compounds were discovered by the means of ADME and CDOCKER. The comp#1, as a representative, was selected to be synthesized and assayed for their CDC25B inhibitory activities. The comp#1 exhibited mild inhibitory activities against human CDC25B with IC_50_ values at about 39.02 μM. Interestingly, MD simulation and MM/PBSA demonstrated that comp#1 bound to the catalytic domain of CDC25B disrupt the protein-protein interaction. In comparison with the existing inhibitor compound 7, the new inhibitors not only had better pharmaceutically relevant properties, but also assumed the conformations more favorable in binding to CDC25B. Hence, based on these studies, the findings here may stimulate a new strategy for developing novel anticancer agents.

## MATERIALS AND METHODS

The representative crystal structures of CDC25B (PDB ID:4WH9) [24] and CDK2/Cyclin A (PDB ID: 5CYI) [30] were downloaded from the Protein Data Bank [31] and used for the following studies.

### Protein-protein docking

ZDOCK associated with RDOCK has been demonstrated as a highly successful method for making accurate protein-protein docking predictions [32]. ZDOCK [25] module of Discovery Studio 3.5 which had displayed great prediction abilities in protein-protein docking was used to perform CDC25B (PDB ID:4WH9) and the interacting regulatory protein CDK2/Cyclin A (PDB ID: 5CYI) docking studies. Initially, proteins were prepared by removing water compounds, adding the hydrogen atoms, assigning bond orders, adding hydrogen, treating metals, treating disulfides utilizing the protocol of “prepare protein” [33, 34]. Then, the key residues Arg 488, Arg 492 and Tyr 497 of CDC25B which was set as “receptor” were considered as receptor binding site residues and Asp 206 and Asp 210 of CDK2/Cyclin A defined as “ligand” were known as ligand binding site residues [27, 28]. It was important to limit the docked surface within the key residues to avoid irrelevant bound complexes (false positives). At last, in the docking process, we set an angular step size to 6°, a root-mean-square deviation (RMSD) cutoff value to 6.0 Å and interface cutoff to 9.0 Å to carry out final conformational sampling. After docking, 2000 top poses were generated and clustered with the maximum number of 60. All possible binding modes between receptor and ligand were ranked based on shape, desolvation energy, and electrostatics using Fast Fourier transform (FFT) algorithm [35]. In order to obtain near-native conformation, the predicted protein poses from ZDOCK were subjected to refinement and re-ranking using CHARMm [36] to remove clashes and optimize polar and charge interactions by RDOCK [26] which consisted of two-stage energy minimization scheme: (1) minimization ionic residues in neutral state [26, 37]; (2) minimization ionic residues at charged state [37]. After refinement, the predictions are rescored based on the combination of electrostatics and desolvation terms and finally re-ranked. E_RDOCK (Energy RDOCK) [26] was used as the default scoring function to predict the binding affinity of the ligand protein to the receptor protein. Hence, the 5 poses of docked conformations which had lower E_RDOCK were selected for further analysis.

### Protein-protein docking validation

In order to avoid false positive results, the validation of docking module is very important procedure. Two methods were performed to assess the reliability of the docking protocol. To start with, the docked structures of the two proteins CDC25B and CDK2/Cyclin A were superimposed with the crystal CDC25B (PDB ID:4WH9) and CDK2/Cyclin A (PDB ID: 5CYI), respectively. The RMSD values between the two structures were both < 3 Å [38], which indicated that the docking method was reliable. It is reported that Arg 488, Arg 492 and Tyr 497 of CDC25B had hydrogen bonding interactions with Asp 206 and Asp 210 of CDK2/Cyclin A, respectively. The distance fluctuated between Arg 488(N1)-Asp 206(OD1) for 0.29 ± 0.02 nm, Arg 492(NE)-Asp 206(OD1) for 0.28 ± 0.02 nm, 0.29 ± 0.02 nm for Arg 492(N1)-Asp 206(OD2) and 0.27 ± 0.02 nm for Tyr 497(OH)-Asp 210(OD1), respectively [27, 28], then, we used it as a standard to verify our model. The 20 ns MD simulations were performed toward 5 poses of the two proteins CDC25B and CDK2/Cyclin A. Four atoms-Arg 488(N1), Arg 492(NE and N1), Tyr 497(OH) in CDC25B and three atoms-Asp 206(OD1 and OD2), Asp 210 (OD1) in CDK2/Cyclin A were monitored and calculated.

### Generation of novel compounds

Fragment replacing was a powerful computational technique which was always applied to discover new inhibitors based on a known inhibitor [39, 40]. Before using this module, high-quality fragment database should be constructed, including two criterions: 1) good diversity to represent drug-like chemical space; 2) meeting certain criteria of physicochemical properties, solubility and synthetic accessibility [41–43]. The database of potential substitution fragments was generated by decomposing over 10 million molecules of Drugs-Now database from ZINC [44] using the module of Discovery Studio called “Generate fragment libraries”. First, The compound 7, which was conceived as a lead compound to develop novel therapeutic agent for treating cancer, was divided into three parts, A, B and C, as marked by blue line, red line and green line, respectively (Figure 7). Second, the three parts of compound 7 were replaced using the module of “replace fragment” by searching the fragment database generated earlier. Lund group reported that the part A formed hydrogen bonds with Phe 386, Leu 398, Lys 399, Cys 484 and Met 505 through water molecules and formed a cation-Pi interaction with Arg 488, the part B was partly solvent exposed and replaced a well-defined water molecule and the part C formed hydrogen bonds with Arg 488 and Arg 492 [24]. Based on this, we replaced part A by searching acidic and aromatic databases to form hydrogen bonds and hydrophobic interactions, while the purpose of replacing part B and C is to form more hydrogen binding interactions with Arg 488 and Arg 492 by searching the whole fragment database.

### ADME prediction

Poor ADME properties cause the failure of many drug candidates during clinical trials. Thus, it was important to exclude those none drug-like compounds before virtual screening. In this study, the means of ADME embedded in Discovery Studio 3.5 was used to evaluate the pharmacokinetic and pharmacodynamic properties of selected compounds, including aqueous solubility [45], human intestinal absorption (HIA) [46, 47], cytochrome P450 2D6 (CYP2D6) binding [48] and plasma protein binding properties (PPB) [49, 50]. The compounds obtained from “replace fragment” were selected based on the values of 2–4, ≤ 1, False and True for solubility, absorption, CYP2D6 and PPB, respectively, which might become promising drugs.

### Virtual screening

CDOCKER [51], a docking algorithm, which is a program used to carry out automated docking ligands to their receptors, was employed to evaluate potential bioactivity of candidate compounds.

The most optimal structure of CDC25B:CDK2/Cyclin A singled out from protein-protein docking was used for docking. Initially protein was prepared by removing water compounds, adding the hydrogen atoms, assigning bond orders, adding hydrogen, treating metals, treating disulfides utilizing the protocol of “prepare protein” [33, 34]. During the process of CDOCKER, the 1st step is to calculate receptor conformations using ChiFlex energy [52]. The 2nd step is to create ligands conformations. The retrieved compounds were subjected to “Prepare Ligand” which consists of the procedures of generating possible states by ionization at target pH 7.0±2.0, desalting, retaining chiralities from 3D structure and geometry minimization with the CHARMm forcefield. The 3rd step is to define the binding site. Binding sphere for CDK2/Cyclin A-bound CDC25B (14.585, -8.852, -2.618 and 11.5) was selected from the active sites using the binding site tool based on the known ligand pose-compound 7. It is a unique binding site targeting the CDC25B and CDK interaction site, primarily including Phe 386, Asp 397, Leu 398, Lys 399, Cys 484, Arg 485, Arg 488, Arg 492 and Met 505 [24], which is not the traditional binding site (PTP domain and swimming pool [53]). The 4th step is to perform ligands docking into the protein active site. The 5th step is to refine selected protein side-chains in the presence of the rigid ligand using ChiRotor [52]. The side chains of specified amino acids in CDC25B (Phe 386, Asp 397, Leu 398, Lys 399, Cys 484, Arg 485, Arg 488, Arg 492 and Met 505) were refined using ChiRotor algorithm. The 6th step is to perform a final ligand refinement using CDOCKER [54]. CDOCKER_ENERGY, as the default scoring function, was applied to predict the binding affinity of the ligand to the target receptor. Hence, the structures which had better CDOCKER_ENERGY than compound7 were selected for further analysis.

### Chemistry

All the reagents were purchased from commercial suppliers and were used without further purification unless otherwise indicated. All the reactions were monitored by thin-layer chromatography (TLC) on silica gel precoated F254 Merck plates, and spots were examined under UV light (254 nm). All column chromatography was performedusing 200–300 mesh silica gel. ^1^H NMR and^13^C NMR spectra were taken on a Bruker Avance 300-MHz NMR Spectrometer at 300 K with TMS as the internal standard, and CDCl_3_ and DMSO-d_6_ were used as solvent, the values of the chemical shifts (δ) were expressed in parts per million (ppm), and coupling constants (*J*) were expressed in hertz (Hz). MS spectra were recorded on an Agilent 1100 LC/MSD (ESI) Mass Spectrum.

### PTP activity assay

Human recombinant CDC25B was expressed in E.coli and purified by Ni-NTA affinity chromatography in our laboratory. The basic chemical reaction catalyzed by a phosphatase converted a phosphosubstrate into a dephosphorylated product and free phosphate which could be measured as a surrogate for phosphatase activity. pNPP (para-nitrophenyl phosphate) was used as phosphatase substrate which can be hydrolyzed by phosphatase to give para-nitrophenol. Subsequently, para-nitrophenol converted into para-nitrophenolate (pNP) with addition of sodium hydroxide stop solution. pNP is an intense yellow compound and could be measured at 405 nm using a spectrophotometer. To begin with, purified recombinant CDC25B (0.05 μg) in 50 μL buffer with 50 mM citrate (pH 6.0), 0.1 M NaCl, 1 mM EDTA, and 1 mM dithiothreitol (DTT) and test compounds were added to each well of a 96-well plate. Blank was prepared by omitting enzyme and substituting an equivalent volume of buffer. After preincubation for 15 min at room temperature, 50 μL of reaction buffer with 2 mM pNPP was added and incubated at 37°C for 30 min. Then, the reaction was stopped by adding 10 μL 0.2 M sodium hydroxide and chilled on ice quickly. In addition, the amount of pNP was measured by detecting the absorption at 405 nm against blank. Finally, IC_50_ values were determined by analyzing the data using ORIGINPRO 8 software.

### Molecular dynamic simulation

The “GROMACS 4.5.5 package” was adopted to study the internal motions of the receptor-ligand system. The interactions of CDC25B-CDK2/Cyclin A with the best hit identified from screening of “Replace Fragment” was investigated through MD simulation using GROMACS 4.5.5 package with GROMACS 43a1 force field [55] for 20 ns. The topology files and charges for the ligand atoms were generated using the PRODRG 4.5.5 Server [56]. All the models were simulated by incorporating space-filling dodecahedron boxes and filled with explicit single-point charge (SPC) water molecules. The models were covered with a water shell of 1.0 nm from the surface of the protein. The system was neutralized and equilibrated with counter ions to replace SPC water molecules randomly. Subsequently, the models were minimized using steepest descent approach and well equilibrated by position-restrained dynamics simulation, namely NVT and NPT canonical ensemble (N= number of partical, P= system pressure, V= volume, and T= temperature). All models were heated to 300 K during a 100 ps NVT simulation with a coupling constant of 0.1 ps and equilibrated with constant pressure of 1 bar in a 100 ps NPT simulation with a coupling constant of 2 ps using leap-frog integrator. The temperature and pressure were regulated by V-rescale, a modified Berendsen thermostat, and Berendsen pressure coupling method, respectively. The short-range electrostatic and van der Waals interactions were cutoff with the radius of 1.5 nm and the long-range electrostatic interactions were calculated by using the Particle Mesh Ewald algorithm [57] with fourth-order cubic interpolation and 0.16 Fourier spacing for both two simulations. All bonds were constrained by using the LINCS algorithm [58]. Finally, all models were performed for duration 20 ns MD simulation and all MD trajectories were recorded every 20 ps with time step of 2.0 fs.

### Binding free energy calculations

Binding free energies for all complex systems were calculated by using the molecular mechanics Poisson Boltzmann surface area (MM-PBSA) method [59]. A total of 50 snapshots that were extracted during equilibrium phase between 10–20 ns were employed for the calculation, using g_mmpbsa [60] tool of Gromacs. The binding free energy of protein with ligand system in solvent was expressed as the following equations [60]:

ΔG_binding_ = G_complex_ - (G_protein_ + G_ligand_)

Here, G_complex_, G_protein_ and G_ligand_ are the total free energies of complex, receptor and ligand in solvent respectively. Furthermore, the free energy for each individual G_complex_, G_protein_ and G_ligand_ was estimated by:

G_x_ = E_MM_ - TS + G_solvation_

E_MM_ = E_bonded_ + E_non-bonded_ = E_bonded_ + (E_vdw_ + E_elec_)

G_solvation_ = G_polar_ + G_non-polar_

Here, x is the protein, ligand or protein-ligand complex. E_MM_ is the average molecular mechanics potential energy in vacuum and G_solvation_ is free energy of salvation. T is the temperature and S is the solute entropy. E_bonded_ is bonded interactions which are made up of bond, angle, dihedral and improper interactions and E_non-bonded_ is non-bonded interactions including van der Waals (Evdw) and electrostatic (Eelec) interactions. ΔE_bonded_ is always taken as zero [61]. G_polar_ and G_non-polar_ refer to the electrostatic and non-electrostatic contributions to the solvation free energy, respectively.
